# Assessment of Cognitive Scales to Examine Memory, Executive Function and Language in Individuals with Down Syndrome: Implications of a 6-month Observational Study

**DOI:** 10.3389/fnbeh.2015.00300

**Published:** 2015-11-18

**Authors:** Xavier Liogier d'Ardhuy, Jamie O. Edgin, Charles Bouis, Susana de Sola, Celia Goeldner, Priya Kishnani, Jana Nöldeke, Sydney Rice, Silvia Sacco, Lisa Squassante, Gail Spiridigliozzi, Jeannie Visootsak, James Heller, Omar Khwaja

**Affiliations:** ^1^F. Hoffmann-La Roche, Roche Pharma Research and Early Development, Neuroscience, Roche Innovation Center BaselBasel, Switzerland; ^2^Department of Psychology, University of ArizonaTucson, AZ, USA; ^3^Research Department, Institut Jérôme LejeuneParis, France; ^4^Cellular and Systems Neurobiology Research Group, Human Pharmacology and Clinical Neurosciences Research Group-Neurosciences Program, Systems Biology Program, Centre for Genomic Regulation, Hospital del Mar Medical Research InstituteBarcelona, Spain; ^5^Medical Genetics, Duke University Medical CenterDurham, NC, USA; ^6^Department of Pediatrics, University of ArizonaTucson, AZ, USA; ^7^F. Hoffmann-La Roche, BiostatisticsBasel, Switzerland; ^8^Department of Pediatrics, Duke University Medical CenterDurham, NC, USA; ^9^F. Hoffmann-La Roche, Roche Pharma Research and Early Development, Neuroscience and Rare Diseases, Roche Innovation Center New YorkNew York, NY, USA; ^10^Formerly of Duke University Medical CenterDurham, NC, USA; ^11^F. Hoffmann-La Roche, Roche Pharma Research and Early Development, Rare Diseases, Roche Innovation Center BaselBasel, Switzerland

**Keywords:** Down syndrome, outcome measure, clinical trial, cognition, language

## Abstract

Down syndrome (DS) is the most commonly identifiable genetic form of intellectual disability. Individuals with DS have considerable deficits in intellectual functioning (i.e., low intellectual quotient, delayed learning and/or impaired language development) and adaptive behavior. Previous pharmacological studies in this population have been limited by a lack of appropriate endpoints that accurately measured change in cognitive and functional abilities. Therefore, the current longitudinal observational study assessed the suitability and reliability of existing cognitive scales to determine which tools would be the most effective in future interventional clinical studies. Subtests of the Repeatable Battery for the Assessment of Neuropsychological Status (RBANS), Cambridge Neuropsychological Test Automated Battery (CANTAB), and Clinical Evaluation of Language Fundamentals-Preschool-2 (CELF-P-2), and the Observer Memory Questionnaire-Parent Form (OMQ-PF), Behavior Rating Inventory of Executive Function®–Preschool Version (BRIEF-P) and Leiter International Performance Scale-Revised were assessed. The results reported here have contributed to the optimization of trial design and endpoint selection for the Phase 2 study of a new selective negative allosteric modulator of the GABA_A_ receptor α5-subtype (Basmisanil), and can be applied to other studies in the DS population.

## Introduction

Down syndrome (DS) is the most common chromosomal cause of intellectual disability (ID). Each year approximately 6000 babies are born in the United States with DS, which is equivalent to 1 in 700 babies (Parker et al., 2010). Worldwide the estimated incidence is approximately 1 in 1000–1100 (World Health Organization (WHO), 2015). DS is characterized by substantial limitations in intellectual functioning (i.e., low intellectual quotient (IQ), delayed learning and/or impaired language development) and adaptive behavior. Studies have revealed a specific neuropsychological profile for this population—individuals typically have an average IQ below 70 (Chapman and Hesketh, 2000; Gioia et al., 2000) and weaknesses consistently associated with associative and verbal working memory (Jarrold et al., 2006, 2008; Silverman, 2007), episodic memory and explicit long-term memory (Carlesimo et al., 1997; Vicari, 2001), expressive language (Miller, 1998), and executive function (Lanfranchi et al., 2010), whereas relative strengths have been observed in visuospatial tasks and implicit long-term memory (Edgin et al., 2010b). Although, IQ levels vary in individuals with DS, most individuals function in the mild to moderate range of ID (Centers for Disease Control Prevention, 2015; Centers for Medicare Medicaid Services, 2014). Of note, as the rate of cognitive development progressively becomes slower over the childhood years in relation to typically developing peers, a decline in IQ scores over the childhood years is also observed (Carr, 1995).

Differences in brain structure and function are already apparent in early infancy in individuals with DS (Nadel, 2003; Edgin et al., 2015), with clear alterations in hippocampus (e.g., altered microarchitecture of pyramidal cells), prefrontal cortex (reduced volume), and cerebellum (e.g., hypoplasia) apparent pre- and post-natally (Pennington et al., 2003; Lott and Dierssen, 2010). Furthermore, structural and volumetric magnetic resonance imaging (MRI) studies have shown that individuals with DS have a smaller intracranial volume than their typically developing peers, with the most profound differences observed in the frontal lobes, cerebellum, and brainstem (Kesslak et al., 1994; Raz et al., 1995; Aylward et al., 1999). Other studies have also shown that smaller volumes are observed in the temporal lobe, including the hippocampal region (Schmidt-Sidor et al., 1990; Pinter et al., 2001) which is known to affect a range of cognitive functions. As individuals with DS approach early adulthood, some are at particular risk for the early development of Alzheimer's disease (Zigman et al., 2008). The prevalence of dementia in DS increases over 45 years of age, with upwards of 75% having dementia over 65 years (Lott and Dierssen, 2010), although neuropathological and neurochemical changes have been observed as early as fetal development (Bahn et al., 2002; de Sola et al., 2015).

Recent advancements in our understanding of the underlying mechanisms of cognitive dysfunction in DS suggest an imbalance between excitatory and inhibitory neurotransmission. Ɣ-Aminobutyric acid (GABA) neurotransmission is the major inhibitory system in the mature brain. Reducing GABA-mediated inhibition by limiting GABA_A_ receptor activity has shown beneficial effects on hippocampal synaptic plasticity as well as learning and memory deficits in the Ts65Dn mouse model of DS (Kleschevnikov et al., 2004; Fernandez et al., 2007; Colas et al., 2013; Martínez-Cué et al., 2013; Potier et al., 2014). A negative allosteric modulator of the GABA_A_ α5-containing receptor subtype (Basmisanil) is currently under investigation in young adults with DS (ClinicalTrials.gov identifier: NCT02024789).

Previous pharmaceutical trials in DS have noted that studies are often limited by a lack of endpoints that accurately captured cognitive and functional changes (Heller et al., 2006). Thus, it is important to assess the suitability and reliability of existing tools that measure cognitive function in a longitudinal observational study to determine which measures may be most effective in the context of a pharmacological clinical trial. Specifically, clinical trials require measures that can be repeatedly and reliably administered across international sites, to participants of a defined age range, and that do not exhibit large practice, floor, or ceiling effects.

The recently published TESDAD battery includes neurocognitive tests and scales, but no test-retest analysis or evaluation of potential practice effect are currently available (de Sola et al., 2015). Edgin et al. also reported the development of the Arizona Cognitive Test Battery (ACTB) based on the Cambridge Neuropsychological Test Automated Battery (CANTAB) and other available tools (Edgin et al., 2010a). The ACTB was designed based on historical findings of performance deficits in domains, and tasks that had been repeatedly shown to be more difficult for those with DS (Pennington et al., 2003; Edgin et al., 2010a, 2014; Lee et al., 2011). The ACTB validation suggested that neuropsychological measures could be administered to a large sample of individuals with DS (*n* = 74) with low floor effects and good preliminary estimates of test-retest reliability (albeit in a small subsample). This battery could have been used in our clinical trials; however, based on the mechanism of action of Basmisanil, some of the tests may be more relevant than others (e.g., hippocampal or prefrontal tests vs. cerebellar function tests). Therefore, alternative scales were chosen for analysis in this study. Furthermore, most measurement validation studies have been limited in their ability to ascertain the reliability of endpoint measures within the retesting time frame and frequency required to determine how the measures perform in a clinical trial context. Given the frequency of new clinical investigations in this population, more measurement development and validation is urgently required, leading us to report on these data to assist the broader community with study design in the future. Furthermore, the National Institutes of Health (NIH) Research Plan on Down Syndrome, which was revised in 2014, reports on the need to study clinical and behavioral treatments and interventions for DS, with part of this plan noting the importance for reliable and valid endpoint assessments to measure the efficacy of these treatments (U.S. Department of Health Human Services National Institutes of Health, 2014).

## Objectives

Given this background, the primary objective of this non-pharmacological study (BP25612; ClinicalTrials.gov identifier: NCT01580384) was to investigate the suitability (i.e., number of participants completing the tests, floor/ceiling effects, and potential learning effect) of selected neurocognitive tests in a 6-month longitudinal and multinational setting for the measurement of cognitive function in individuals with DS. Subtests of the Repeatable Battery for the Assessment of Neuropsychological Status (RBANS) (Randolph et al., 1998), subtests of CANTAB (Cantab Research Suite, 2015), subtests from the Clinical Evaluation of Language Fundamentals-Preschool-2 (CELF-P-2) (Pearson, 2004), the Observer Memory Questionnaire-Parent Form (OMQ-PF) (Gonzalez et al., 2008), and the Behavior Rating Inventory of Executive Function®–Preschool Version (BRIEF-P) (Gioia et al., 2000) were used to assess immediate and delayed memory, language, and executive function. Secondary objectives were to assess the test-retest reliability of these measures over 6 months and to explore the influence of age (adolescents vs. adults) and non-verbal IQ level, as measured by the Leiter International Performance Scale-Revised (Leiter-R) (Roid and Miller, 1997).

Part of the results from this study were previously presented at the 2014 American Association of Intellectual and Developmental Disabilities (AAIDD) Annual Meeting (del Valle Rubido et al., 2014), as well as at the 2013 Cognition in Down Syndrome Workshop (Liogier d'Ardhuy et al., 2013). Results from the assessments using the Vineland Adaptive Behavior Scales-II (VABS-II) and the Clinician Global Impression of Severity (CGI-S) and Improvement (CGI-I) scales will be reported separately.

## Methods

This was a 6-month (24–27 weeks) observational, non-pharmacological, longitudinal, multicenter (11 sites), multinational study in adolescents (12–17 years) and adults (18–30 years) with DS conducted between February 2012 and January 2014. The study was conducted in the United States, United Kingdom, Spain, France, Italy, Canada, and Argentina. Overall 90 participants (equally split between adolescents and adults) were planned to be enrolled and randomized into three different schedules of assessments (i.e., A, B, and C; C contained a smaller number of tests and visits). In order to include all of the planned assessments and keep the duration within the desired 90-min testing period for each study visit, three schedules of assessments were implemented. A 15–25 min break was planned after 45 min of testing and an additional break could be added before starting the last exercise (RBANS) if requested or deemed necessary by the rater. Randomization was stratified by age group to have a balanced number of sequences of assessments between adolescents and adults.

The current study was conducted for 6 months to reflect the clinical trial design of the ongoing Phase 2 study. Participants who met the inclusion criteria (below) received testing at the baseline visit, 4 weeks and 24 weeks later when randomized to schedule A or B or received testing at the baseline visit and at 24 weeks when randomized to schedule C (Table 1). These schedules resulted in a common data set that was administered to at least 60 participants. The total duration of the study for each participant was between 24 and 27 weeks.

**Table 1:** **Number of participants per randomization schedule and total number of subjects evaluated per task**.

**Scale**	**Subscale**	**Schedule**			**Total number of participants**
		**A**	**B**	**C**	
Leiter-R		30	30	30	90
CANTAB	SSP	30	30		60
CELF-P-2		30	30	30	90
RBANS	List learning	30	30	30	90
	Story memory	30	30	30	90
	Picture naming	30	30	30	90
	Semantic fluency	30	30	30	90
OMQ-PF		30	30	30	90
BRIEF-P		30	30		60

*Randomization was stratified by age, with an equal number of participants in the 12–17 and 18–30 years age groups. For schedules A and B assessments were done at baseline, week 4 and week 24. For schedule C assessments were done at baseline and week 24 only*.

### Study population

Male and female adolescents (12–17 years) and adults (18–30 years) with a diagnosis of DS were included in the study if they met all of the following criteria: parent/caregiver was able to speak and understand the local language, to accompany the participant to all clinic visits, and to provide information about the participant's behavior and daily functioning. Also, the participant's speech was understandable to the examiner; at screening the participant attempted to perform the neuropsychological tests; stable treatment for at least 8 weeks prior to screening if he/she had a generalized anxiety disorder, major depressive disorder, autism spectrum disorder, attention-deficit/hyperactivity disorder, and recent laboratory tests confirming euthyroid (serum free thyroxine [FT4] and thyroid stimulating hormone [TSH]) and normoglycemic (serum glucose) status (within 12 months prior to screening visit, with or without treatment). Individuals were not included if they met any of the following criteria: diagnosed with axis I and II psychiatric disorders, except those mentioned above; exhibited significant suicidal risk; could not comply with protocol or perform the outcome measures due to hearing or visual impairment; had evidence of dementia; had thyroid dysfunction or diabetes not adequately controlled at least 8 weeks prior to randomization; or abused alcohol and/or other substances.

Written informed consent was obtained from the parents/caregivers and assent from the participants prior to participation in the study. The study was conducted in accordance with the principles of the Declaration of Helsinki and Good Clinical Practice (GCP), and all required approvals were obtained from the appropriate independent ethics committee (IEC)/institutional review board (IRB) prior to the start of the study.

### Concomitant medication

Psychotropic agents that would likely interfere with any of the assessments could not be initiated or changed during the study period. This included antidepressants (e.g., selective serotonin reuptake inhibitors [SSRIs], serotonin and norepinephrine reuptake inhibitors [SNRIs], norepinephrine-dopamine reuptake inhibitors such as bupropion, and serotonin-norepinephrine reuptake inhibitors such as the tricyclic antidepressants), antipsychotics, benzodiazepines and hypnotics, acetylcholinesterase inhibitors, GABA agonists (e.g., tiagabine, vigabatrin, and baclofen), and glutamatergic drugs (e.g., riluzole, topiramate, memantine, and lamotrigine).

### Procedures

Selected raters for the cognitive assessments/rating scales were provided with instructions and comprehensive training on scale administration prior to the start of the study. Whenever possible, for each participant the same rater/caregiver consistently administered/completed the rating scales across study visits.

The assessments were completed in a prespecified and consistent order to maximize standardization across sites and participants.

### Scales selected to measure cognitive skills

#### The Leiter international performance scale-revised (Leiter-R) (Roid and Miller, 1997)

Leiter-R, a non-verbal intelligence test, was individually administered to all participants. Two reasoning subtests (Sequential Order and Repeated Patterns) and two visualization subtests (Figure Ground and Form Completion) were administered to derive a non-verbal IQ.

#### Repeatable battery for the assessment of neuropsychological status (RBANS) (Randolph et al., 1998)

The RBANS was individually administered and used to measure cognitive changes over time. Four subtests of the full battery of 12 subtests were used in this study to assess immediate memory (List Learning and Story Memory), as well as language capacities (i.e., Picture Naming and Semantic Fluency). The RBANS was chosen because it has been used in clinical trials investigations (Duff et al., 2010; Hobson et al., 2010) and provides four alternate forms. Alternate forms were used on each study day. The raw score on each of these scales was used for analysis.

#### Cambridge neuropsychological test automated battery (CANTAB) (CANTAB Research Suite, 2015)

The CANTAB is a computerized battery of neuropsychological tests carried out by the participant under the supervision of qualified personnel. The Spatial Span (SSP) subtest was used in this study to assess working memory capacities; it is considered a visuospatial analog of a digit span test in which a random array of boxes on a screen change color in a particular sequence. The participant's response was given by recalling the test pattern in forward or reverse order.

#### Observer memory questionnaire-parent form (OMQ-PF) (Gonzalez et al., 2008)

The OMQ-PF is a 27-item questionnaire designed to ascertain the perceptions of parents/caregivers about the participant's daily memory function. It has been previously validated in children with temporal lobe epilepsy and memory impairment (Gonzalez et al., 2008). Items were rated on a 5-point Likert scale (1- strongly agree to 5- strongly disagree OR 1- never to 5- always).

#### Behavior rating inventory of executive function®–preschool version (BRIEF-P) (Gioia et al., 2000)

The BRIEF-P was completed by the parent/caregiver and measured the participant's everyday skills associated with executive function (i.e., Inhibit, Working Memory, Plan/Organize, and the Global Executive Composite [GEC]). This scale has been used in a number of investigations of DS, where it demonstrated a unique pattern of strengths and weaknesses, including deficits in parent's ratings of working memory and planning, but not in inhibition or emotional control (Lee et al., 2011).

#### Clinical evaluation of language Fundamentals-Preschool-2 (CELF-P-2) (Pearson, 2004)

The CELF-P-2 consists of a variety of subtests used to evaluate the language skills of preschool-aged children (3–6 years). The Word Classes subtest was used to evaluate the participant's ability to understand and express relationships between semantically related words. Raw scores from the receptive and expressive scales of this subtest were used in the analyses.

The CELF-P-2, RBANS, and OMQ-PF were translated into French, Italian, and Spanish by a process that included forward translation, back translation, and concept validation. Rater instructions for the Leiter-R and CANTAB were also translated. The BRIEF-P was already available in various languages and did not require translation for use in this study.

### Statistical methods

For the assessments with a minimum of 60 participants, a Mixed Model Repeated Measurements (MMRM) analysis was applied with visit-time as repeat factor; subject as subject-effect; gender, language and age as class factors; age by visit-time as interaction; and baseline IQ as continuous covariate. Estimates of the mean differences between age groups, genders and visits (6 months vs. baseline), and the estimate of the slope (β) over IQ were derived.

Measurements of between-subject variability and residual variability as well as of correlation between repeated assessments within the same subjects were extracted from the mixed model. As a measure of test-retest reliability, Intraclass Correlation Coefficient (ICC) was derived per each age group between visits (6 months vs. baseline). An ICC was considered poor, fair, good, and very good when values were < 0.40, 0.40–0.59, 0.60–0.75, and >0.75, respectively (Cicchetti and Sparrow, 1981; Oremus et al., 2012). Analyses of correlation at baseline were performed between RBANS List Learning and both CELF-P-2 Expressive and OMQ-PF scores, and between CANTAB SSP reverse and BRIEF-P scores (i.e., GEC and Working Memory subdomains).

All derived *p*-values were not controlled for multiple comparisons and should be interpreted as an aid to gauge the magnitude of estimated differences.

## Results

### Study population

A total of 94 participants were screened, 90 were randomized (49 adolescents 12–17 years; 41 young adults 18–30 years), and 89 completed the study; the participant who did not complete the study was lost to follow up. Table 2 shows the study demographics. The mean age for the adolescent and adult groups was 15 years and 23 years, respectively. The adult group was well balanced for gender (51% female, 49% male), whereas slightly more males were enrolled in the adolescent group (59%). No procedure-related adverse events (e.g., fatigue or tiredness) were recorded in any participants.

**Table 2:** **Study demographics**.

	**12–17 years**	**18–30 years**
*N*	49	41
Females	20 (41%)	21 (51%)
Males	29 (59%)	20 (49%)
**AGE**		
Mean ± SD	14.5 ± 1.6	22.7 ± 3.4
Median	15	22
Range	12–17	18–30
**IQ (LEITER-R)**		
Mean ± SD	41.6 ± 7.1	39.0 ± 6.0
Mean (F/M)	39.9/42.7	40.4/37.6
Range	36–80	36–65
**COUNTRY (*****N*****)**		
Argentina	7	3
Canada	2	6
France	17	6
Italy	6	5
Spain	11	6
UK	0	2
US	6	13

### Neurocognitive assessments

The baseline IQ scores are shown in Table 2. The mean IQ scores were similar between age groups (adolescents 42 ± 7; adults 39 ± 6), although 22% of adolescents and 61% of adults performed at the floor (36) of the test (Table 3).

**Table 3:** **Test-retest reliability (ICC) between baseline and 6 months and floor effect at baseline**.

		**Leiter-R**	**BRIEF-P (composite)**	**CELF-P-2 (expressive)**	**CELF-P-2 (receptive)**	**CANTAB SSP length FW**	**CANTAB SSP length REV**	**OMQ-PF**	**RBANS Picture Naming**	**RBANS Semantic Fluency**	**RBANS List Learning**	**RBANS Story Memory**
Adolescents	ICC	NA	**0.78**	**0.71**	**0.63**	**0.67**	0.53	**0.76**	0.50	0.59	**0.69**	**0.69**
	Floor^*^	11/49 (22%)	NA	7/49 (14%)	1/49 (2%)	1/34 (3%)	8/34 (24%)	NA	5/49 (10%)	3/49 (6%)	2/49 (4%)	11/49 (22%)
Adults	ICC	NA	**0.77**	**0.69**	**0.68**	0.55	0.40	**0.90**	0.53	**0.73**	**0.64**	**0.67**
	Floor^*^	25/41 (61%)	NA	3/41 (7%)	1/41 (2%)	0 (0%)	6/27 (22%)	NA	2/41 (5%)	3/41 (7%)	3/41 (7%)	5/41 (12%)

* *Floor: number of subjects at the lowest possible value of the assessment over the total number of subjects assessed. Bold values correspond to ICC ≥ 0.60 (good)*.

### Memory assessments

#### RBANS (list learning and story memory)

The List Learning baseline scores followed a relatively normal distribution, ranging from 0 to 32, over a maximum possible score of 40, with means of 11.8 (standard deviation [SD] 7.5) and 13.8 (SD 8.2) for the adolescents and adults, respectively (Table 4). Very few participants had a score of zero in this task (Table 3; 4 and 7% for adolescents and adults, respectively). However, 24% of adolescents and 12% of adults had very low scores (≤ 4). The average reference List Learning scores for typically developing individuals aged 20–39 years is approximately 30 (Randolph, 2006). Overall, adults had statistically higher List Learning scores than adolescents (age, *p* = 0.035; Table 5). The adolescents showed improvement (+2.3 ± 5.6) over the 6-month period, whereas the adults did not, as captured by the close to significant time x age interaction. The IQ scores were significantly related to the List Learning scores (*p* < 0.001; Table 5).

**Table 4:** **Mean scores and standard deviations at baseline and 6 months for each scale**.

**Mean ± SD (*N*)**		**12–17 years**	**18–30 years**
RBANS list learning	Baseline	11.8 ± 7.5 (49)	13.8 ± 8.2 (41)
	6 Months	14.1 ± 7.3 (49)	13.8 ± 7.0 (39)
RBANS story memory	Baseline	6.0 ± 5.2 (49)	5.6 ± 4.1 (41)
	6 Months	4.4 ± 3.4 (49)	5.5 ± 4.3 (39)
RBANS picture naming	Baseline	6.3 ± 2.3 (49)	6.4 ± 2.1 (41)
	6 Months	6.4 ± 2.4 (49)	6.7 ± 2.7 (39)
RBANS semantic fluency	Baseline	8.1 ± 5.1 (41)	7.2 ± 3.7 (49)
	6 Months	6.6 ± 4.1 (39)	6.3 ± 3.0 (49)
CANTAB SSP length (forward)	Baseline	3.5 ± 1.0 (27)	3.2 ± 1.1 (33)
	6 Months	3.6 ± 0.9 (26)	3.3 ± 1.3 (33)
CANTAB SSP length (reverse)	Baseline	2.5 ± 1.7 (27)	2.2 ± 1.4 (33)
	6 Months	2.8 ± 1.2 (26)	2.2 ± 1.4 (33)
CELF-P-2 (expressive)	Baseline	9.0 ± 5.6 (49)	12.9 ± 5.7 (41)
	6 Months	10.5 ± 5.5 (49)	12.0 ± 6.5 (39)
CELF-P-2 (receptive)	Baseline	14.5 ± 4.8 (49)	16.3 ± 4.6 (41)
	6 Months	14.8 ± 4.6 (49)	15.7 ± 6.0 (39)
BRIEF-P (composite)	Baseline	104 ± 16.4 (34)	92.1 ± 20.9 (27)
	6 Months	101 ± 16.0 (34)	91.2 ± 20.8 (26)
OMQ-PF	Baseline	94.4 ± 13.0 (49)	99.1 ± 13.0 (34)
	6 Months	93.9 ± 17.0 (48)	100 ± 14.3 (37)

**Table 5:** **Estimates of differences for each assessment and influence of Time, Age, and IQ (***p***-values as from MMRM analysis)**.

	**BRIEF-P (composite)**	**CELF-P-2 (expressive)**	**CELF-P-2 (receptive)**	**CANTAB SSP length FW**	**CANTAB SSP length REV**	**OMQ-PF**	**RBANS Picture naming**	**RBANS Semantic fluency**	**RBANS List learning**	**RBANS Story memory**
**ESTIMATES**										
6 Months –Baseline	−1.79	0.39	−0.08	0.16	0.25	0.21	0.16	−1.17	1.20	−0.81
Adults –Adolescents	−13.42	2.15	1.16	0.43	0.77	6.09	0.30	0.87	3.17	1.03
Females—Males	−0.63	2.51	1.30	−0.22	−0.25	2.80	0.71	1.82	2.09	0.74
**TESTS OF EFFECTS**										
Time	0.291	0.373	0.844	0.147	0.155	0.824	0.513	<**0.001**	0.051	**0.025**
Age	**0.011**	0.056	0.261	0.095	**0.019**	0.075	0.519	0.297	**0.035**	0.250
Time × Age	0.684	**0.014**	0.387	0.814	0.435	0.387	0.752	0.417	0.078	**0.030**
IQ	0.931	**0.003**	**0.024**	<**0.001**	**0.001**	0.331	**0.005**	**0.006**	<**0.001**	**0.001**

*Bold values correspond to p < 0.05*.

Overall, the Story Memory scores ranged from 0 to 21 out of a maximum possible score of 24 with means of 5.6 (SD 4.1) and 6.0 (SD 5.2) for the adults and adolescents, respectively (Table 4). The distribution was skewed toward the lower scores, illustrating a floor effect. This was particularly evident in the adolescent group, with 22% obtaining a score of 0 at baseline, reflecting the difficulty of this subtest for this population. However, on average, both age groups performed equally in the Story Memory subtest (*p* = 0.250; Table 4). Adolescents scores decreased on average over the 6-month period (−1.6 ± 3.5 SD), whereas adult scores did not change over time (time × age, *p* = 0.030; Table 5). IQ scores were significantly related to the Story Memory scores (*p* = 0.001; Table 5).

#### OMQ-PF (daily memory)

The baseline distributions of total raw scores for both age groups appeared normal, ranging from 61 to 124 (reference for typically developing children 5–16 years of age, 107). There was no significant difference in the observed memory scores between age groups (6.09, *p* = 0.075) or visits (0.21, *p* = 0.824; Table 5). IQ level did not predict perceived daily memory scores. The observed memory score correlated with the RBANS List Learning score across ages (*r* = 0.33, *p* < 0.01), demonstrating concurrent validity with a direct memory assessment.

### Executive function assessments

#### CANTAB (spatial span)

For the forward span length, the baseline distribution was normal in both age groups and no floor effect was observed (Table 5). On the other hand, in the reverse task, 24% of the adolescents and 22% of the adults scored 0. On average, adults had significantly greater reverse span length (+0.77, *p* = 0.019; Table 5), whereas no difference was observed between age groups for the forward span performance (age, *p* = 0.095). Forward and reverse span lengths were stable over time (age × time 0.814 and 0.435, respectively; Table 5). IQ was related to both forward (*p* < 0.001) and reverse (*p* = 0.001) span length (Table 5).

#### BRIEF-P

At baseline, the BRIEF-P GEC scores in the adolescent group were normally distributed, whereas the adult group peaked at lower values (better). Adults had statistically lower mean BRIEF-P GEC scores compared with adolescent (−13.42, *p* = 0.011), indicating higher perceived executive functioning in this age group. GEC scores were stable across visits (time, *p* = 0.291).

IQ was not related to GEC scores (*p* = 0.931, Table 5). To further explore this lack of influence of IQ, correlations between IQ scores and the Working Memory domain, the Plan/Organize and the Inhibit domains were conducted and did not show any relation, in either age group. No significant correlations were found between BRIEF GEC scores and either forward or reverse span lengths from the CANTAB SSP tasks. Nevertheless, BRIEF-P Working Memory scores correlated with reverse SSP length (*R* = −0.27, *p* = 0.036, moderate effect).

### Language assessments

#### RBANS (picture naming and semantic fluency)

The baseline distribution of scores for both subtests followed normal distribution for both age groups, and a small number of participants performed at the floor of the tests (Table 3). No age differences were detected. Whereas, no effect of time was noticed in the Picture Naming task, time had a significant effect on Semantic Fluency results with lower scores at 6 months than at baseline (-1.17, *p* < 0.001, Table 5). Both Picture Naming and Semantic Fluency scores were significantly related to IQ (*p* = 0.005 and *p* = 0.006, respectively).

#### CELF-P-2 (linguistic functioning)

**Figure 1:**
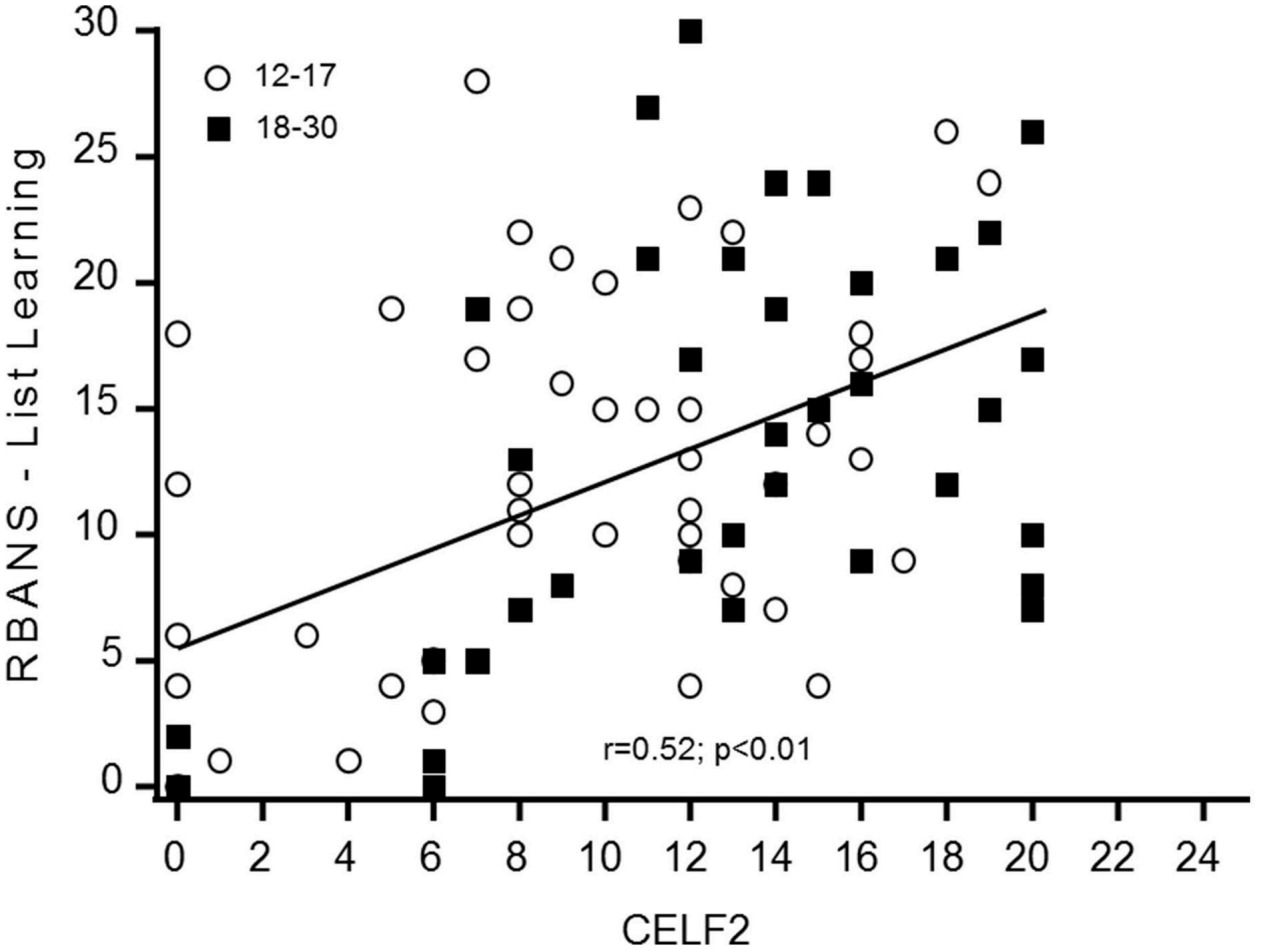
**RBANS List Learning scores and CELF-P-2 Word Classes expressive scores**.

The baseline distribution of total scores in the CELF-P-2 was normal for the adolescents but was skewed toward the higher values for adults. This is likely due to a significant number of adult participants (*n* = 12) reaching the maximum score (or close to) of 20 for the receptive domain (but not for the expressive). Of note, female participants had a statistically higher average total scores (+3.95, *p* = 0.037) and expressive scores (+2.51, *p* = 0.016) than males. No gender differences were observed in receptive scores, likely due to the ceiling effect in this domain. Time did not affect any of the CELF-P-2 sub-scores. The total CELF-P-2 scores were significantly related to IQ scores (*p* = 0.001), driven by both the expressive and the receptive domains (*p* = 0.003; *p* = 0.024, respectively). To better understand the minimum level of language skills required to perform key cognitive tasks, we tested for correlations between receptive and expressive components of the CELF-P-2 and the RBANS List Learning and Semantic Fluency scores. In both age groups, CELF-P-2 expressive scores highly correlated with RBANS Semantic Fluency scores (*p* < 0.001) and with RBANS List Learning scores (Figure 1).

### Test-retest reliability

A summary of ICCs for all scales is shown in Table 3. Reliability ranged from fair (ICC 0.40–0.59) to very good (ICC > 0.75). Most of the scales depicted good reliability (ICC = 0.63: CELF-P-2, RBANS Semantic Fluency, List Learning and Story Memory subtests, BRIEF-P and OMQ-PF). The highest ICC scores were found for the BRIEF-P and OMQ-PF, which are both parent-reported scales.

## Discussion

This study assessed a variety of neurocognitive tests and functioning scales over a 6-month period to determine appropriate outcome measures for potential use in interventional pharmacological and non-pharmacological treatment studies in adolescents and young adults with DS. To date, this is the largest data set reporting evaluation of these assessments.

**Figure 2:**
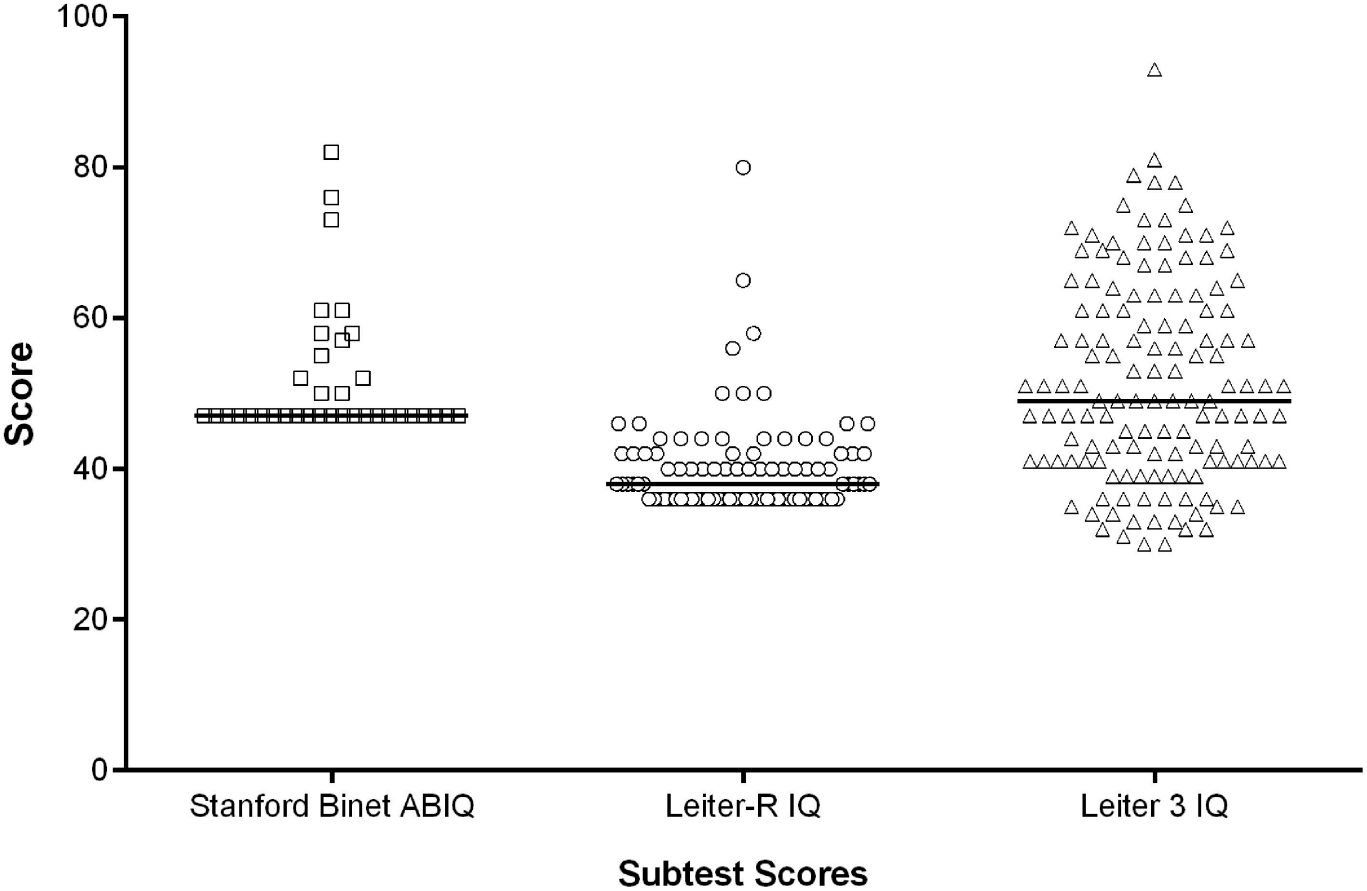
**IQ scores: distributions using three different IQ scales**. The horizontal bar represents the median score.

The Leiter-R IQ scale is a non-verbal assessment that is not influenced by linguistic production which is particularly impaired in individuals with DS. Moreover, in an international clinical trial context, form equivalence after language translation is a major barrier to the implementation of IQ scales. The Leiter-R is not influenced by this issue. Our results show that the Leiter-R may not be the most suitable means of capturing the lower end of the IQ range in DS as 22% of adolescents and 61% of adults scored at the floor of the test (36); however, this test has shown better results than those obtained in a previous clinical trial with the abbreviated Stanford-Binet Intelligence Scales Fifth Edition (ClinicalTrials.gov Identifier: NCT01436955). Based on these observations, the Leiter-3 (Roid and Miller, 1997) was administered in a study with 180 adults and adolescents with DS (Clinical.Trials.gov Identifier NCT01920633). These results are more promising in terms of data distribution and percentage of participants at the floor of 30 (approximately 1%). This suggests that the Leiter-3 is probably more appropriate to measure the full IQ range in this population (Figure 2). In studies in children with DS, it is not uncommon for standardized IQ scores to decrease across childhood (Carr, 1995). In our study of older individuals with DS (12–30 years), using the Leiter-R we found stability in IQ scores similar to the recent findings by Carr, showing no change in IQ from 21 to 45 years in a longitudinally collected sample(Carr, 2012). However, with the greater number of adults at the floor of 36, any age-related differences may have been masked by floor effects.

The RBANS was developed for the dual purposes of identifying and characterizing abnormal cognitive decline in older adults and as a neuropsychological screening battery for younger patients (Randolph et al., 1998). With average List Learning scores of 14 for adults with DS where the average score in typically developing peers is approximately 30, and even greater discrepancies in the Story Memory subtest, this demonstrates that these tasks are very difficult for individuals with DS. Some improvements in performance were observed over the 6-month study period and, in particular, adolescents showed improvement in the List Learning task. These observations may be linked to the natural neurodevelopment of the capacities of adolescents and/or the fact that more adolescents with DS are attending school and involved in alternative therapies such as speech therapies and educational resources. The Story Memory scores, however, did not show a similar improvement in adolescents which may be due to a greater floor effect.

Observed memory is not a direct measure of the participant's memory capacities, but a functional measure that can be affected by many facets of mnemonic ability in daily life. Overall, the OMQ-PF showed good reliability and suitability for use in clinical trials of individuals with DS. Previous results by Gonzales et al. have indicated that the OMQ-PF may be more closely related to new learning ability rather than retention or recall of information (Gonzalez et al., 2008), and other studies suggest that everyday abilities cannot necessarily be inferred from direct neuropsychological tasks (Chaytor and Schmitter-Edgecombe, 2003). Our results suggest that observed memory scores relate to specific memory functions, as illustrated by the correlation noted between the RBANS List Learning immediate and OMQ-PF scores.

Forward and backward SSP subtests were administered to assess working memory. Here we recapitulate the known working memory deficit in DS (Lanfranchi et al., 2012), with low scores in both forward and reverse tasks and a significant number of participants scoring 0, or “floor” effects, in the reverse task. Both subtests were statistically related to IQ scores, however, this relationship is likely driven by the floor effects in both IQ and spatial span tests, and thus less meaningful. Neither forward nor reverse SSP length correlated with BRIEF-P GEC scores. Overall, these findings together with low ICC values indicate that SSP would be too difficult and discouraging for individuals with DS and have limited usefulness as an outcome measure in interventional clinical trials.

The BRIEF-P was implemented as an indirect measure of executive function, including working memory function. Here again executive function deficits were clear, confirming the neurocognitive DS profile. An obvious difference was evident between the adolescent and adult groups in GEC scores, with adults performing significantly better than adolescents. Adult performance reached maximum scores, suggesting that the preschool version of the BRIEF is probably less appropriate for the adults than the adolescents with DS. The BRIEF-school age version (5–18 years) could have been used instead. This version of the BRIEF was indeed used as a behavioral assessment to establish concurrent validity for the ACTB (Edgin et al., 2010a). The perceived global executive function was not influenced by IQ across ages. We therefore looked at IQ correlations in adolescents and adults separately in BRIEF-P subdomains and interestingly noted that neither, the Working Memory, Plan/Organize or Inhibit subtests correlated with IQ. However, a focused analysis of Working Memory aspects, considered to be a major contributor to executive function weaknesses in DS, revealed that the Working Memory domain of the BRIEF-P correlated with reverse SSP, a direct Working Memory executive function measure. These findings suggest that the BRIEF-P captures executive functions engaged in the reverse SSP processing, but overall distinct functions than those captured by the Leiter.

Language difficulties are one of the most prominent barriers to independence and socialization and part of the neurocognitive profile in DS. Here we assessed elements of linguistic functioning. The CELF-P-2 Word Classes test showed a potential “ceiling” effect, reducing its use to assess changes in language abilities in a trial; nevertheless, the link between CELF-P-2 expressive scores and RBANS List Learning performances suggests this test could be of relevant use as a screening tool in future studies to ensure enrolment of participants with the minimal level of expressive language ability required to perform key cognitive tasks. In our study, the verbal communication level was on average better in females as compared to males, particularly in the expressive domain, as assessed by the CELF-P-2 Word Classes and RBANS Semantic Fluency, confirming the previously described communication profile in DS (Määttä et al., 2006).

Language proficiency was also tested with the Picture Naming and Semantic Fluency tasks from the RBANS. Overall, the test-retest scores from these two tests were considered fair, illustrating a potential lack of suitability for clinical trials in individuals with DS. However, to avoid potential practice effects, four different RBANS forms have been developed to be used on several occasions in clinical trials. A weakness in our study is that the same RBANS form was used at the baseline visit but two different forms were used at the Week 24 visit depending on the study schedule. This might explained the low ICC scores that we observed or the time effect observed in the Semantic Fluency task.

Finally, we observed that direct measurements of immediate memory, executive function and linguistic functioning as described here, were all influenced by the IQ level of the participants. On the other hand, indirect measures of executive function and memory as reported by the parents or the caregivers (BRIEF-P and OMQ-PF) were not sensitive to the IQ level.

Table 6 summarizes the main findings for each scale evaluated in this study and our conclusions on their suitability for clinical trials with adults and adolescents with Down syndrome. These conclusions contributed to the selection of suitable outcome measures for the ongoing 26-week Phase 2 study (Clinicaltrials.gov identifier: NCT02024789) evaluating the efficacy, safety and tolerability of Basmisanil in individuals (12–30 years) with DS. RBANS List Learning was chosen as the primary endpoint for evaluating hippocampal tasks associated with a global functioning evaluation, whereas the Leiter-3 was selected as the IQ measure. These results can be relevant to other trials assessing cognitive function in the DS population, but also in other conditions. Given the breath of these measures we have validated scales that could be used across trials, including memory interventions (RBANS, OMQ-PF) as well as in attention deficits (BRIEF-P, CANTAB spatial span).

**Table 6:** **Summary of key learnings**.

**Domain**	**Test name**	**Suitable for clinical trials with people with DS**		**Summary of main findings**
		**12–17 years**	**18–30 years**	
IQ measurement	Leiter-R	No	No	- Floor effect observed at 36
	Leiter-3	Yes	Yes	- No floor effect, good distribution
Memory	RBANS—Short term memory			- Differences between the forms
	List learning	Yes	Yes	- Good test-retest reliability, no floor effect, sensitive to age and IQ
	Story memory	No	Yes	- Floor effects and unstable over time in adolescents.
	OMQ-PF	Yes	Yes	- Good stability over time and good test-retest reliability
Executive function	CANTAB SSP			
	Forward	Yes	Yes	- No floor effect, good test-retest reliability, sensitive to IQ
	Reverse	No	No	- Floor effects in both age groups, low reliability
	BRIEF-P	Yes	No	- Reliable, stable and sensitive to age and detects impairment in the working memory domain
				- Ceiling effect in adults
Language	CELF-P-2 Word classes	Yes	Yes	- Stable, reliable and sensitive to age and IQ
				- Ceiling effect in the receptive domain in adults (recommend to use CELF-4)
	RBANS			
	Semantic fluency	Yes	Yes	- No floor effect, sensitive to spoken language and IQ but not age
	Picture naming	No	No	- Low test-retest reliability

## Conclusion

To our knowledge, the results reported here are the first from a multinational study assessing cognitive function in a substantial number of adolescents and adults with DS over a 6-month period, allowing both robust suitability and reliability analyses. Multiple assessments that evaluate overlapping cognitive functions were conducted, which allowed for a robust characterization of these scales and their interrelationships. Finally, these findings provide information on the natural neurocognitive changes in adolescents and adults with DS over a 6-month period, which will contribute to a better understanding of the true impact of intervention in future efficacy trials.

Overall, the current study has important implications for measuring cognitive changes in response to pharmacological treatment. Such non-pharmacological, longitudinal studies are key in the development of medicine for neurodevelopmental disorders such as DS where the choice of appropriate tools is critical to be able to detect beneficial drug effects.

### Conflict of interest statement

Xavier Liogier d'Ardhuy, Celia Goeldner, Jana Nöldeke, Lisa Squassante, Jeannie Visootsak, Omar Khwaja are employed by F. Hoffmann-La Roche. Jamie O. Edgin received grants from F. Hoffmann-La Roche during the conduct of the study, personal fees from F. Hoffmann-La Roche and Novartis outside of the submitted work, and has a patent pending. Priya Kishnani received personal fees/non-financial support from F. Hoffmann-La Roche during the conduct of the study. Gail Spiridigliozzi received grants from F. Hoffmann-La Roche during the conduct of the study and outside of the submitted work. Charles Bouis, Susana de Sola, Sydney Rice, and Silvia Sacco declare that the research was conducted in the absence of any commercial or financial relationships that could be construed as a potential conflict of interest.
